# Perspective – ultrastructural analyses reflect the effects of sleep and sleep loss on neuronal cell biology

**DOI:** 10.1093/sleep/zsac047

**Published:** 2022-02-28

**Authors:** Lijing Wang, Sara J Aton

**Affiliations:** Department of Molecular, Cellular, and Developmental Biology, University of Michigan, Ann Arbor, MI, USA

**Keywords:** sleep, sleep deprivation, organelle, ultrastructure, heterochromatin, Golgi, endoplasmic reticulum (ER), endosomes, lysosomes, reactive oxygen species, unfolded protein response

## Abstract

Recent electron microscopic analyses of neurons in the *Drosophila* and rodent brain demonstrate that acute or chronic sleep loss can alter the structures of various organelles, including mitochondria, nucleus, and Golgi apparatus. Here, we discuss these findings in the context of biochemical findings from the sleep deprived brain, to clarify how these morphological changes may related to altered organelle function. We discuss how, taken together, the available data suggest that sleep loss (particularly chronic sleep loss) disrupts such fundamental cellular processes as transcription, translation, intracellular transport, and metabolism. A better understanding of these effects will have broad implications for understanding the biological importance of sleep, and the relationship of sleep loss to neuropathology.

**Statement of Significance**

Since the earliest applications of microscopy to the nervous system, neuronal structure has provided clues to neuron and circuit function. Here we discuss how recent observations of how sleep loss affects organelle structures in neuronal somata, and how these structural changes may reflect underlying cell biological changes within the brain. Together, observed structural and biochemical changes are providing new insights into how sleep loss affects the function of neurons at the cellular level.

Sleep loss affects brain function in numerous ways, including disrupting both working and long-term memory, attention, and decision making. While the last two decades have provided new insights into how sleep loss affects neural activity [1–5], gene expression [6, 7], and protein translation [8-11], a complete understanding of the cell biological effects of sleep deprivation (SD) in the brain is still lacking. A recent study by Flores et al. in *Sleep* [12] addresses this question using serial block-face electron microscopy (SBEM) in a *Drosophila* brain structure involved in long-term memory storage. Kenyon cells are intrinsic neurons that integrate a variety of inputs within the mushroom bodies – an associative learning center analogous to the mammalian hippocampus. Previous work has shown that a few hours of brief SD increases spontaneous activity and olfactory responses in Kenyon cells [13]; this is associated with structural changes to Kenyon cells’ synaptic outputs indicating both decreases and increases in synaptic strength [14]. Critically, however, longer-duration SD (24 h or more) suppresses Kenyon cell activity and makes sensory responses unreliable [13]. To better understand the intracellular effects of prolonged SD on Kenyon cells, Flores et al. used SBEM to reconstruct their cell bodies and intracellular organelles after ad lib sleep, 11-h SD, and 35-h SD. This study built on previous work from the lab, using single-plane electron microscopy (EM) to resolve these structures in adolescent mouse prefrontal cortex [15]. The results of that work suggested that mitochondrial size and density within pyramidal neurons’ cell bodies was increased by chronic (multiple days) sleep disruption, and to a lesser extent, by acute (a few hours) sleep deprivation (Figure 1). Sleep loss also led to changes in the densities of pyramidal neurons’ lysosomes and early endosomes, suggesting subtle changes to intracellular trafficking [15].

**Figure 1.**
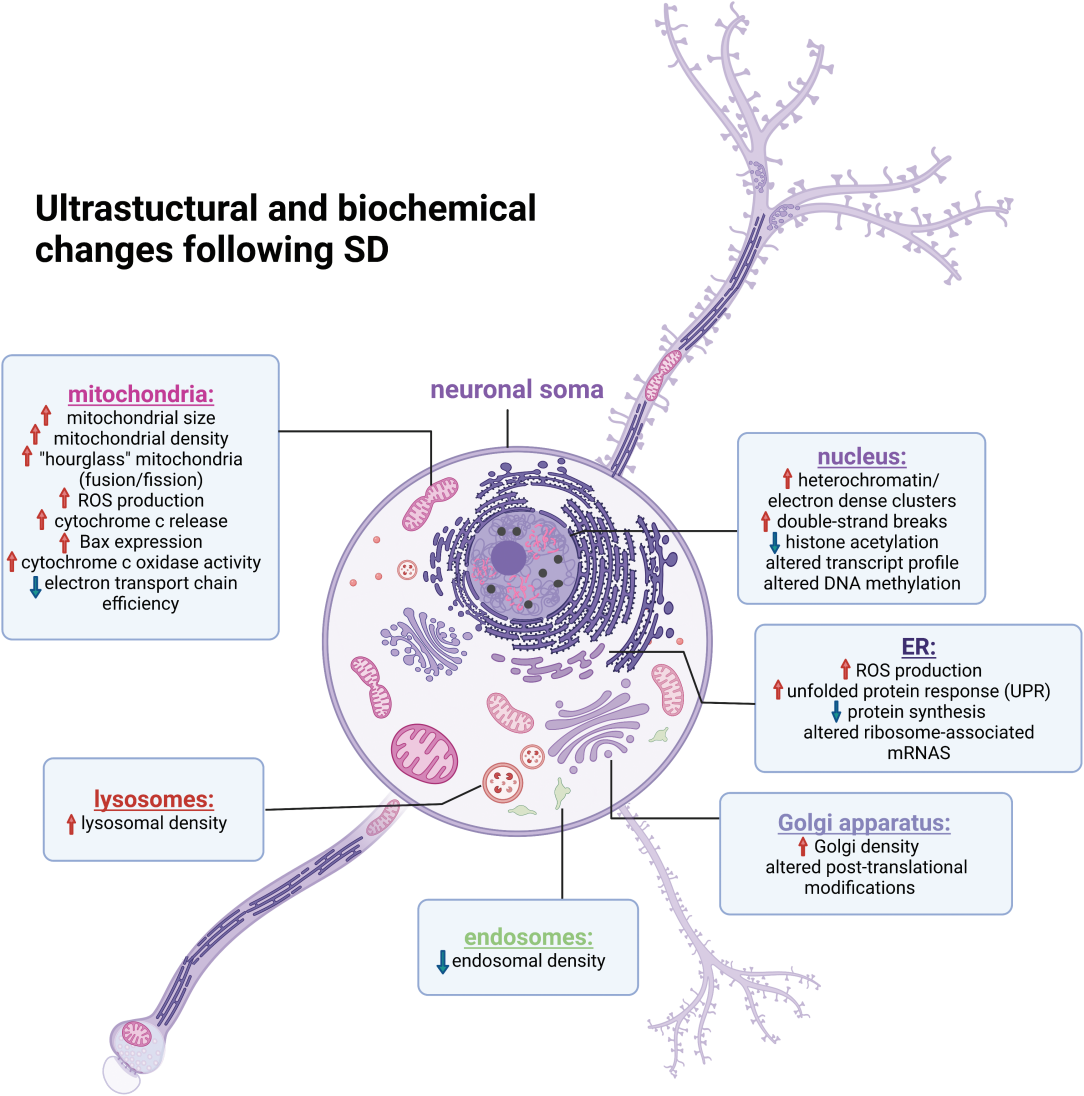
Summary of both morphological and biochemical alterations to neuronal organelles following SD. While many organelles such as mitochondria, Golgi, and ER are also present in axons and dendrites, it is unclear how morphology changes in those structures with SD. It is also unclear whether biochemical/functional changes to organelles are cell compartment-specific. Figure was created with BioRender.com.

Because the conclusions that can be drawn from single-plane EM are necessarily limited, Flores et al. now use full reconstruction of Kenyon cell bodies in the adult *Drosophila* brain, using serial block-face scanning electron microscopy (SBEM), to characterize cell structures following either ad lib sleep, 11-h SD, or 35-h SD. The authors quantified changes to organelles in both nucleus and cytoplasm components following SD (Figure 1). Consistent with the lab’s prior findings in mouse neocortex [15], total Kenyon cell cytoplasmic volume occupied by mitochondria showed a non-significant trend for increasing with increasing-duration sleep loss. The density of Golgi apparatus showed a similar (non-significant) trend, with a tendency for higher Golgi numbers per cell in 35-h SD flies than freely-sleeping flies. More prolonged (i.e. 35-h) SD was also associated with a (non-significant) trend for smaller nuclear volume and nucleus-to-cytoplasm volumetric ratio. Within the nucleus, the authors found statistically significant increases in the absolute number and density of electron-dense “dark clusters” of chromatin following SD of both durations. Among all the measures made by the authors, dark cluster density was the most important parameter for discriminating between Kenyon cells from flies in the three treatment groups.

Here we discuss what these findings could mean, in the context of other recent data, for our understanding of how SD affects the basic cellular biology of neurons in the brain. We also discuss some questions raised by these data that need to be answered in order to understand how SD affects neuronal function.

## Effects of Sleep Loss on Neuronal Nuclear Function

The reported increase in dark, roundish nuclear clusters in Kenyon cells suggests possible epigenetic changes to neurons as a function of sleep loss. A similar phenomenon was recently reported using EM – with electron-dense clusters observed in rat hippocampal neurons’ nuclei after chronic (14-day) sleep restriction, with or without caffeine administration [16]. Although the exact driver of cluster formation is still unclear, such clusters likely reflect the same process of heterochromatin formation observed in mammals in the context of both cellular senescence [17] and neuronal plasticity [18]. The increased number and density of heterochromatin clusters after longer-duration SD suggests that long-term sleep loss may disrupt patterns of transcriptionally-active DNA in neurons. Several lines of evidence support this idea (Figure 1). For example, one recent study using an assay for transposase-accessible chromatin with sequencing (ATAC-seq) to identify transcriptionally-accessible regions of DNA showed that SD rapidly (within as little as 3 h) and dramatically changes chromatin accessibility in the mouse cerebral cortex. Surprisingly, however, the vast majority of the differentially accessible DNA sites showed increased, rather than decreased, accessibility after SD [19] – which is more typical of euchromatin. SD may also affect transcription by altering DNA methylation. Studies using methylated DNA immunoprecipitation in rat cortex [20] and 5 mC and 5 hmC arrays in mouse cortex [21] have demonstrated altered methylation patterns after as little as 3-6 h of SD, which are associated with bidirectional changes in gene transcription. SD may also affect histone acetylation, which increases DNA accessibility to transcription factors. Recent findings suggest that both acute and chronic SD increase histone deacetylase activity, reducing levels of histone H3 and H4 acetylation in mouse and rat brain [22–24]. Treating animals with histone deacetylase inhibitors rescued both SD-induced cognitive disruption [22, 24] and late long-term potentiation (LTP) impairments caused by SD [22]. Histone deacetylation could drive the formation of heterochromatin, and thus the formation of electron-dense nuclear clusters reported by Flores et al. and Xie et al. [12, 16].

However, another possibility is that these dark clusters reflect sites of DNA damage, such as those occurring during senescence [17]. It is thought that heterochromatin regulation after DNA damage is important for silencing damaged genes, maintaining genomic stability [25]. Prolonged wake induces double-strand breaks in DNA within *Drosophila* neurons (Figure 1), and SD can disrupt repair of these breaks [26]. More recent work in zebrafish has shown that sleep facilitates neuronal chromosome rearrangements essential for DNA repair, while SD disrupts these mechanisms [27]. One possibility is that DNA damage accumulation during SD (through generation of reactive oxygen species; ROS [28, 29], or other mechanisms) ultimately leads to neuronal senescence and cell death. Indeed, recent data suggest that neurodegeneration can be triggered in the mammalian brain through this SD-driven mechanism [30].

Thus, a critical unanswered question from the Flores et al. study centers on clarifying the precise nature of these electron-dense nuclear clusters. Understanding how their formation reflects DNA damage, repair, and transcriptional regulation will be essential for understanding how SD affects the most fundamental cell biological processes of neurons – i.e. central dogma (DNA→mRNA→protein) and cell survival.

## Sleep Deprivation, Cellular Energetics, and Mitochondrial Function

Mitochondria produce energy for the brain in the form of ATP. This bioenergetic function is regulated by sleep-wake cycles, with higher levels of ATP in wake-active regions of the brain during spontaneous sleep, and ATP reduction during SD [31]. As a byproduct of ATP production, mitochondria generate ROS. Alterations in mitochondrial function could thus couple sleep loss to oxidative stress-mediated DNA damage. Multiple studies across species have found that ROS are generated at higher levels in the brain during prolonged SD [32–34] (Figure 1). This is despite (or perhaps, due to) the fact that prolonged sleep disruption reduces efficiency of the ATP-generating mitochondrial electron transport chain. Critically, this reduction persists in some regions of the brain even after the opportunity for recovery sleep [35]. In turn, generation of ROS in mitochondria is directly coupled to sleep homeostatic responses in sleep regulating neurons in the *Drosophila* brain [29, 32].

Beyond the effects of SD on mitochondrial energetics, long-term sleep disruption has also been shown to increase Bax expression in mitochondria in the hippocampus, and release of cytochrome c from mitochondria into the cytoplasm [36] (Figure 1). These changes have been linked to both reduced excitability in hippocampal neurons (likely due to reduced ATP [36]) and initiation of neuronal apoptosis and neurodegeneration [37].

Available data suggest that at least in the first hours of sleep loss, neuronal mitochondria respond to this energetic challenge in several ways. Mitochondria in the mammalian neocortex show upregulation of cytochrome c oxidase expression and activity [38–40] and antioxidant responses [33] after 3–12 h of SD (Figure 1). The morphological changes described in Flores et al. after more prolonged SD in flies [12], and described in de Vivo et al. in mice [15], may also be an adaptive response to SD-driven disruption of mitochondrial metabolic function. One possibility is increasing mitochondrial abundance in neuronal somata is essential for neurons to survive SD-induced disruption of electron transport chain efficiency. This process could be driven by *de novo* organelle biogenesis [41], mitochondrial transportation to the soma from other cellular compartments (i.e. neurites) [42], or increased mitochondrial biogenesis through fission [43]. The recent report of an increased proportion of “hourglass”-shaped mitochondria after SD in mouse pyramidal neurons [15] (Figure 1) suggests that either mitochondrial fission or fusion may be enhanced by sleep loss. Reported increases in “hourglass”-shaped mitochondria, the presence of “extra-large” mitochondria [15], and a trend toward higher proportion of hyperfused mitochondria [12] after prolonged SD may also reflect the formation of so-called “megamitochondria” through membrane fusion. Megamitochondrial formation likely reflects a process aimed at combating the unfavorable cellular environments and decreasing intracellular ROS level [44] following acute or prolonged SD. While both fission and fusion can be adaptive cellular responses to mitochondrial stress, it is important to note that both are also an essential feature of apoptosis [44, 45].

Other morphological changes to neuronal mitochondria themselves have been reported after prolonged SD – e.g. decreased relative volume of intercristal space, which have been reported in both rat hippocampus and neocortex [46]. These intra-organelle morphological changes may relate to SD-driven changes in mitochondrial cristae functions – e.g. changes in cytochrome c storage or electron transport chain activity [46–48]. Taken together, the reported effects of SD on mitochondrial morphology suggest a major impact of sleep loss on neuronal energy production, and potentially also on neuronal viability.

Neuronal proteostasis, and intraneuronal transport – effects of sleep loss on Golgi, endoplasmic reticulum (ER), endosomes, and lysosomes

Flores et al. report a non-significant trend for increased density of Golgi apparatus per Kenyon cell body after longer-duration SD [12]. While de Vivo et al. did not directly measure Golgi or ER, they did report significant increases in pyramidal neurons’ lysosomal size and density with acute and chronic SD, respectively, and reductions in endosomal density with chronic SD [15]. Because the ER, Golgi, lysosomes, and endosomes mediate membrane-associated protein production, trafficking, and quality control, together these ultrastructural findings could reflect SD-induced alterations to intracellular transport and proteostasis.

Across species, biochemical [8, 49–52], transcriptomic [6, 53], and ribosome profiling [8, 11] data suggest that protein translation/quality control and transport are affected by acute sleep loss. For example, in nematodes, *Drosophila*, and mice [49, 51], a brief period of SD leads to an enhanced unfolded protein response (UPR; the cellular stress response to accumulation of misfolded protein in the ER lumen) (Figure 1). Early SD-induced UPR effects in the brain include suppression of protein translation [9], post-translational modification [54], and increased expression of molecular chaperones [52, 55, 56]. While these changes may aid in normalizing protein quality under conditions of cellular stress, sustained UPR activation engages pro-apoptotic pathways [57, 58], ultimately leading to neurodegeneration.

Disruption of ER function also impacts nuclear function. Critically, another site of potentially DNA-damaging ROS production in neurons is the ER, where ROS are made in the process of chaperone-assisted protein folding [59, 60]. Thus, one possibility is that some of the increased ROS observed in the brain with SD are generated by changes to biochemical processes within the ER (Figure 1).

Changes in Golgi density, lysosomal size and density, and endosome density after SD all suggest that neuronal transport and quality control of membrane-associated protein cargo may change with sleep loss. Critically, however, analysis of these structures within the cell body alone cannot give a complete picture of how sleep loss affects protein synthesis, quality control, and transport. Moreover, to date, there is no data on how sleep loss affects the subcellular distribution of the Golgi, ER, lysosomes, or endosomes in neurons. Appropriate subcellular localization and organization of these organelles is essential for spatial regulation of both protein and mRNA, which in neurons plays vital roles in neurotransmission, synaptic plasticity, and information storage [8, 61, 62]. Beyond these essential functions, the presence of Golgi and ER in neurites plays additional roles, including local calcium buffering, regulation of synaptic extracellular glycoproteins, and lipid biogenesis [63, 64]. A recent study from our lab, using neuronal compartment-specific ribosome profiling, demonstrates that mRNAs translated in these membrane-bound organelles vary dramatically with both prior learning and subsequent sleep or SD [8]. Understanding how sleep and SD affect intraneuronal movement of these organelles and their functions in neurites will be essential to understanding how sleep and sleep loss affect neuronal cell biology.

## Future Directions

The findings of Flores et al. provide new insights into how cellular structures are affected by sleep loss in Kenyon cells, which play an essential role in *Drosophila* cognitive function. As is true with transcriptomic and biochemical responses to sleep loss, available data suggest that these changes may also be conserved in neurons across species. This raises a biologically important question – what do these structural changes indicate with respect to the physiological and metabolic processes occurring within neurons? It will be vital for future studies to explore the relationships between the morphological changes present in neuronal organelles after SD, the biochemical and physiological changes occurring within those organelles, and the neuronal and brain-level functional changes due to sleep disruption. Moreover, studies of how organelle morphology, function, and transport within axonal and dendritic cell compartments are affected by sleep and SD will be vital to clarify how brain states affect the cell biology of these essential neuronal structures. Finally, further work is needed to understand (1) what aspects of sleep and SD control these basic mechanisms and (2) how neuronal cellular changes affect the brain and cognition. Progress on these fronts should yield clues to essential, evolutionarily-conserved, sleep functions.
